# A Meta-Analysis of Higher-risk Myelodysplastic Syndrome Trials to Evaluate the Relationship between Short-term Endpoints and Overall Survival

**DOI:** 10.7150/jca.33175

**Published:** 2019-08-29

**Authors:** Abhinav Kurumaddali, Ahmed Hamed Salem, Suresh K. Agarwal

**Affiliations:** 1 Department of Pharmaceutics, University of Florida, Gainesville, FL; 2 Abbvie, Inc., North Chicago, IL; 3 Department of Clinical Pharmacy, Faculty of Pharmacy, Ain Shams University, Cairo, Egypt

**Keywords:** Myelodysplastic syndrome, surrogate endpoints, overall survival, response rates, azacitidine, treatment-naive

## Abstract

**Background**: The objective of this work was to evaluate the relationship between the response rates and median overall survival (OS) in higher-risk myelodysplastic syndrome (HR-MDS) to determine whether response rates could be used as predictors of median OS.

**Methods**: Relevant MDS clinical trials were identified through a review of published literature. Weighted linear regression was performed with various linearizing transformations of response rates and median OS using the in-house built HR-MDS clinical trials database. Covariates of interest were evaluated using a forward inclusion, backward elimination covariate model building procedure at α=0.01 and α=0.005, respectively.

**Results**: Twenty-five trials involving 38 cohorts were included in the meta-analysis. The analysis demonstrated that partial response (PR) or better rate (sum of complete response (CR), marrow complete response (mCR) and PR rates) was a strong predictor of median OS (adjusted R^2^=0.64). The median OS was 3.3 months longer (P < 0.005) with azacitidine treatment compared to treatment with other drugs for a given response rate and prior therapy status. We also have shown that the median OS of treatment naïve HR-MDS patients was 4.5 months longer (P < 0.0001) compared to that of previously treated patients for a given response rate and treatment group.

**Conclusion**: Significant correlation between PR or better rate and median OS in HR-MDS highlights the potential to use PR or better rate as a surrogate endpoint to accelerate development of novel therapies for MDS.

## Introduction

Overall survival (OS) is the universally accepted standard primary endpoint for the approval of oncology drugs 1. This is considered as the most reliable cancer endpoint since it can be measured easily, precisely, and is devoid of subjective bias. However, its evaluation requires long-term patient follow-up and may be influenced by various confounding factors such as crossover or sequential therapies, change in disease severity, or disease progression in the study population before death etc., increasing the time and resources required for the clinical development of novel therapies. In some hematological malignancies, short-term response rate-based outcomes such as complete response rate (CRR) or overall response rate (ORR) can be used as surrogate endpoints, which can lead to expedited clinical drug development of novel therapies via accelerated drug approval pathway by regulatory agencies. From 1992 to 2017, the United States Food and Drug Administration (US FDA) granted accelerated approval of oncology drugs for 93 new indications, 87% of which were based on response rates as surrogate endpoints 2. It is worth mentioning that over the past decade 34 (15%) of the 226 US FDA granted regular approvals for new indications of oncology drugs were based on response rate as the end-point, highlighting the importance of short-term end points in the clinical development of oncology drugs 3.

Currently, only two drugs, azacitidine and decitabine, which act as hypomethylating agents have been approved in the United States for the treatment of newly diagnosed and secondary higher-risk myelodysplastic syndrome (HR-MDS). In addition, azacitidine and decitabine are also currently recommended by the National Comprehensive Cancer Network (NCCN) guidelines for HR-MDS patients who are ineligible for allogenic stem cell transplant therapy. The relationship between response rates and OS has not been assessed across HR-MDS clinical trials, although trends have been observed in individual studies. In a recent Phase 2 study evaluating the combination of lenalidomide and azacitidine for patients with higher-risk MDS, 16 (44%) of 36 patients with complete response (CR) had much higher median overall survival (OS) of 37+ months compared to the median OS of only 13.6 months for the entire cohort 4. In another clinical study investigating the efficacy of intravenous clofarabine for higher-risk MDS, median OS was 21.7 months for complete responders, which was significantly higher compared to only 7.4 months for the entire cohort, indicating a positive correlation between median OS and response rates 5. In the AZA-001 trial, a Phase 3 trial reported in 2009, investigating azacitidine vs. conventional care regimen in HR-MDS patients, OS was positively correlated with CR and partial response (PR). In azacitidine treated group, where 30 (17%) and 21 (12%) of the 179 patients achieved CR and PR responses, respectively, median Kaplan-Meier (K-M) OS was 24.5 months (9.9 - not reached) at the time of last follow-up (median follow-up of 21.1 months [IQR 15.1 - 26.9]). On the other hand, in the conventional care regimen group, where only 14 (8%) and 7 (4%) of the 179 patients achieved CR and PR responses, respectively, median K-M OS was 15 months 6. However, a subsequent analysis of the trial data revealed that azacitidine prolonged the OS even in patients who were not responders 7, 8. Hence, to investigate if short-term response rates are correlated to overall survival in HR-MDS patients, we conducted a systematic meta-analysis of available published MDS clinical trials.

## Methods

### Trial Selection

The primary source of information for this database was PubMed articles published in English between the years 2008 and 2017. During the PubMed search, the patient sub filters used were "myelodysplastic syndrome," "treatment-naïve," “pre-treated,” “hypomethylating agent failure” and '"azacitidine, decitabine or lenalidomide," and the study design and publication type sub-filters were "clinical trial," "monotherapy," "combination therapy," and "primary publication." Azacitidine, decitabine and lenalidomide were chosen, as these drugs are currently recommended by FDA for use in MDS patients who are unfit for allogenic stem cell transplantation and conventional chemotherapy. Azacitidine (75 mg/m^2^ daily for 7 days administered subcutaneously or through IV infusion) and decitabine (15 mg/m^2^ by IV infusion over 3 hours repeated every 8 hours for 3 days or 20 mg/m^2^ over 1 hour repeated daily for 5 days) are the two nucleoside metabolic inhibitors used as first line of therapy for HR-MDS patients. A trial was included in the database if at least one cohort of the study had at least one primary or secondary outcome reported as OS or response rate. Other information that was collected includes trial design, sample size, treatment, MDS type, prior therapy status of patients, percentage of males, age, baseline bone marrow blast percent, percent of higher-risk MDS patients and the IWG criteria (2000 vs. 2006) used for assessing the response rates, if reported 21, 22. A trial was excluded from the database if the study type was not relevant, including retrospective studies, reviews, meta-analyses, case-reports or cost-analyses.

### Meta-analysis Methodology

Descriptive statistics were performed to summarize the characteristics of the cohorts selected for the analysis. To improve the linearity of the relationship, various transformations of response rates (e.g. logit, arcsine) and median OS data (e.g. logarithm, reciprocal, square root, and cube root) were tested. Linear regression weighted by sample size was then performed to determine the correlation between response rates: complete response (CR), marrow complete response (mCR) or better, partial response (PR) or better, hematological improvement (HI) or better and median OS using R v.3.4.1 (http://www.r-project.org/). Effects of covariates such as median age, percentage of males enrolled, treatment, type of therapy (monotherapy vs combination therapy), population (< 30% AML patients vs ≥ 30% AML patients), prior treatment status (treatment naïve vs pre-treated patients) and the IWG criteria (2000 vs 2006) were evaluated using a stepwise forward inclusion, backward elimination model building procedure at Type 1 error rates of α=0.01 and α=0.005, respectively. Adjusted coefficient of determination (R^2^) and model diagnostic plots were used to assess the performance of the models.

## Results

**Figure 1:**
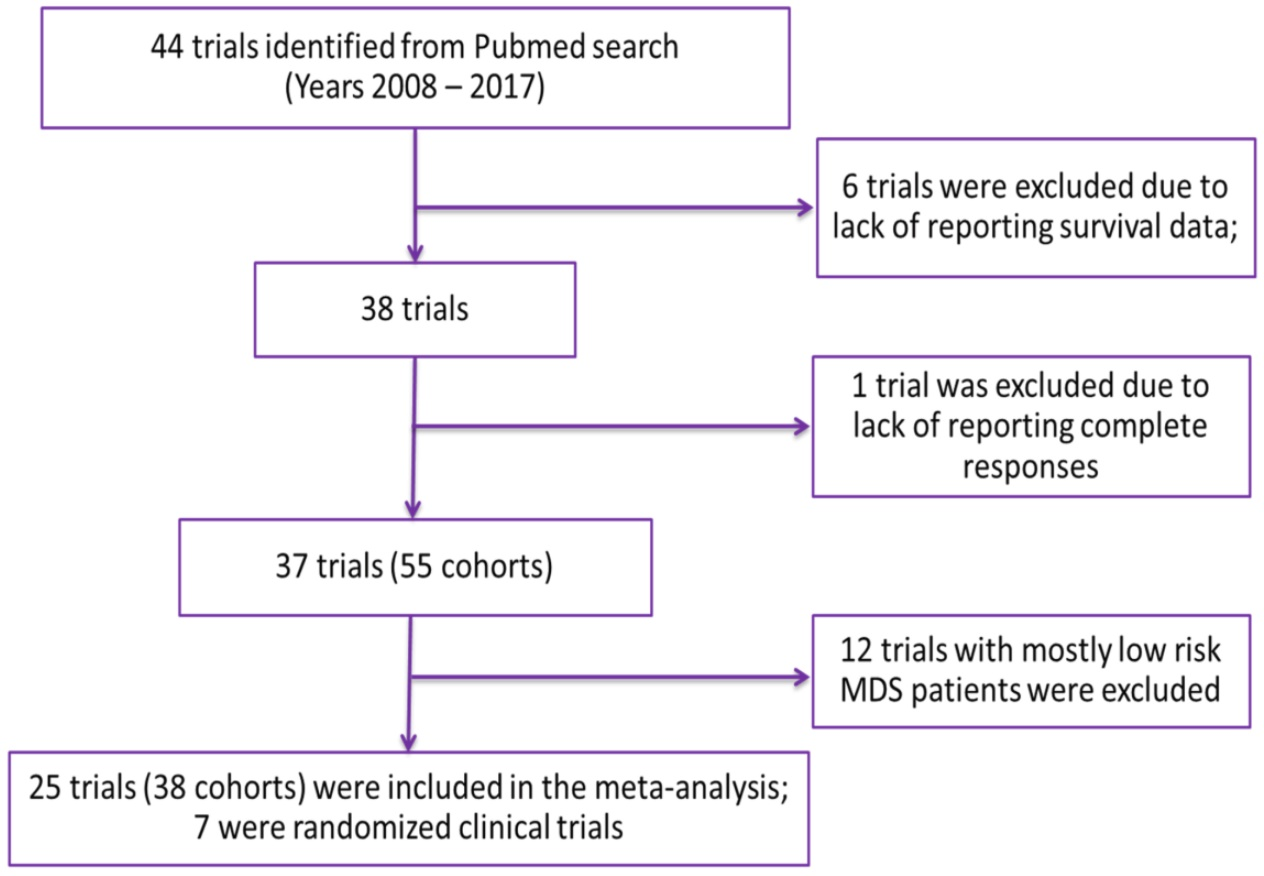
Selection of Trials for Analysis

Overall, 44 trials were included in the database following the outlined inclusion and exclusion criteria. Using the established database, a trial was included in the meta-analysis if it had both response rates and median OS reported in at least one cohort of the study (Figure 1). Twenty-five trials (seven of which were randomized) involving 38 cohorts were included in the meta-analysis. A detailed description of characteristics of cohorts included in the analysis is presented in Table 1. In brief, the median age of patients ranged from 52 to 77.5 years and the median and range of outcomes were as follows: CR 14.9% (0-46.9%); mCR 5% (0-81%); PR 0% (0-25%); HI 5.2% (0-36%) and OS 13.1 months (3.1-24.5 months). Forty-two percent of the cohorts included azacitidine alone or in combination, with lenalidomide being the second most used treatment option (15.8%). Median age was not reported for one cohort (2.6%) and the median percentage of males was not reported for seven cohorts (18.4%). The missing median age was imputed with a median value of 70 years, which was imputed for cohorts in trials not reporting median age. The median percentage of males ranged from 43% to 80% with a median value of 64% imputed for cohorts in trials in which it was not reported.

### Meta-analysis

None of the transformations significantly improved the linear relationship. Therefore, both response rates and median OS were used as untransformed (on original scale) in the final model. The linear regression coefficient was used to assess the strength of the relationship between response rates (CR, mCR or better, PR or better and HI or better) and median OS. The correlation between PR or better and median OS was higher (adjusted R^2^ = 0.64) than that of CR and median OS (adjusted R^2^=0.58). Although the correlation between mCR or better or HI or better (adjusted R^2^=0.64 and 0.70 respectively) was slightly higher compared to the correlation between PR or better and median OS, this was based on a smaller number of studies. This is because 4 cohorts and 4 trials that did not report the HI and mCR values respectively were excluded while determining the relationship between mCR or better or HI or better and median OS. Therefore, the model with PR or better was chosen as the final model.

**Figure 2:**
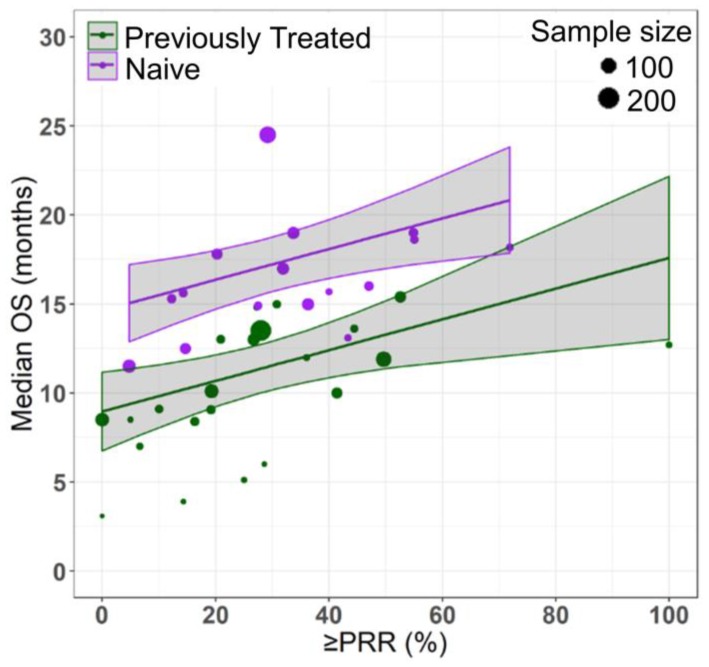
Relationship between partial response rate (PRR) or better and median overall survival (median OS) in HR-MDS with prior treatment status as a covariate. Each filled circle corresponds to a treatment cohort, with the area of the circle being proportional to its sample size. Purple lines indicate the fitted values for treatment-naive cohorts and green lines indicate fitted values for cohorts with previously treated patients. Shaded regions indicate the 95% confidence intervals.

**Figure 3:**
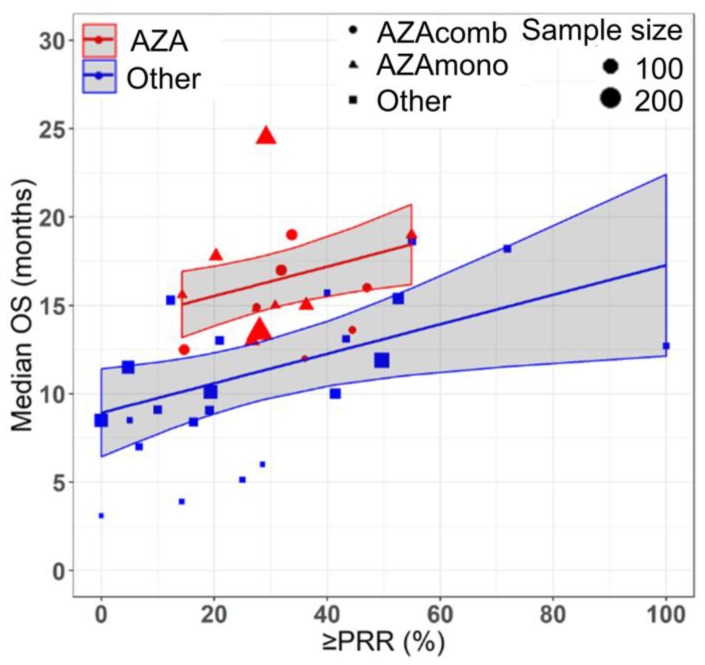
Relationship between partial response rate (PRR) or better and median overall survival (median OS) in HR-MDS with drug treatment as a covariate. Each filled shape (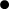 AZAcomb; 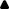 AZAmono; 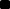 Other) corresponds to a treatment cohort, with the area of the shape being proportional to its sample size. Red lines indicate the fitted values for azacitidine cohorts and blue lines indicate fitted values for cohorts treated with other drugs. Shaded regions indicate the 95% confidence intervals.

**Figure 4:**
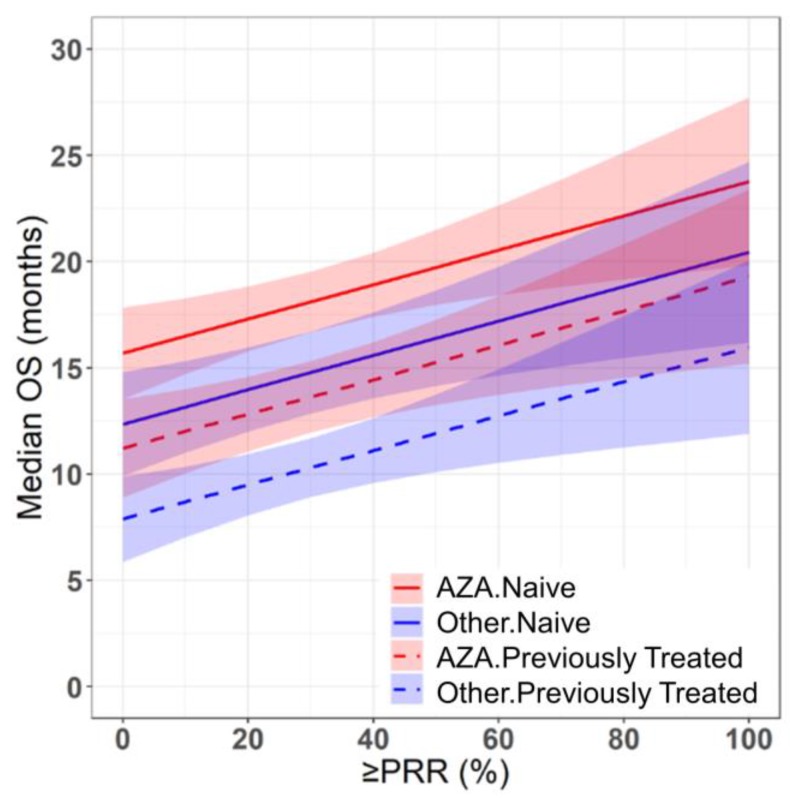
Prediction of median overall survival (median OS) from partial response rate (PRR) or better in HR-MDS using the final model with drug treatment and prior treatment status as covariates. Solid and dashed red lines indicate the predictions for treatment naïve and previously treated azacitidine cohorts respectively. Solid and dashed blue lines indicate the predictions for treatment naïve and previously treated non-azacitidine cohorts respectively. Shaded regions indicate the 95% confidence intervals.

Azacitidine treatment was found to be a significant covariate in the model (P < 0.005), with higher median OS in cohorts receiving treatment with azacitidine at a given PR or better rate compared with those receiving other treatments. For example, at a PR or better rate of 30%, the estimated median OS was approximately 3.3 months (95% CI: 1.0 - 5.6 months) longer in treatment-naïve patients for the azacitidine cohort compared to the non-azacitidine cohorts (Figure 2). Prior treatment status was also found to be a significant covariate in the model (P < 0.0001), with higher median OS in cohorts of predominantly treatment naïve patients at a given PR or better rate compared with cohorts of predominantly previously treated patients. For example, at a PR or better rate of 30%, the estimated median OS was approximately 4.5 months (95% CI: 2.4 - 6.6 months) longer in patients on azacitidine therapy for the treatment naïve cohorts compared to the previously treated cohorts (Figure 3). The final model showed significant correlation between PR or better rate and median OS (R^2^=0.64, Figures 2 - 4). No statistically significant interaction was found between the two covariates, treatment and prior treatment status (Figure 4). Other covariates, such as median age, percentage of males, type of therapy (combination vs monotherapy), population (< 30% AML patients vs ≥ 30% AML patients) and the IWG criteria (2000 vs 2006) were not found to be statistically significant.

## Discussion

The current work represents the first evaluation of the use of response rates as predictors of median OS in MDS, potentially accelerating the development of novel HR-MDS therapies. Findings from this work show that short-term response rates of PR or better can serve as surrogate markers for OS in HR-MDS patients. There is an unmet medical need for better therapies for HR-MDS patients; however, the use of OS as an endpoint may require 2-3 years to demonstrate survival benefit. On the other hand, response rates usually require only 8-10 months follow up in MDS trials. Hence, the use of response rates as surrogate measures for establishing efficacy in MDS trials has the potential to accelerate drug approval by a few years 9, 10.

To speed up the availability of drugs for treating serious diseases, US FDA has developed four distinct pathways: priority review, breakthrough therapy, accelerated approval and fast track apart from the regular approval pathway. For diseases like MDS with an unmet medical need, accelerated approval can be granted based on a surrogate endpoint such as response rates, which can subsequently be converted to a regular approval upon demonstration of direct clinical benefit or an effect on the primary endpoint such as median OS. Generally, in leukemia, durable CR has been used as an endpoint for regular approval of novel therapies if CR was associated with less infection, decreased transfusion requirements and increased median OS 1. At the individual patient level, Komrokji et al. showed that CR was positively correlated with OS and recommended that the CR by IWG 2006 response criteria can be used as a surrogate endpoint for OS for regulatory purposes 11. However, the Phase 3 AZA-001 trial revealed that azacitidine prolonged the OS even in patients who were not responders, suggesting remission does not always result in survival benefit and vice-versa 7, 8. Therefore, there is considerable interest in understanding the relationship between less stringent responses and OS and factors affecting this relationship in elderly patients. To that end, our analysis demonstrated that OS was strongly correlated with PR or better rate in elderly HR-MDS patients.

Along with other drugs, the analysis included clinical trials that evaluated the two commonly used drugs (azacitidine and decitabine) that are currently recommended by FDA and NCCN guidelines for use in elderly patients with HR-MDS who are unfit for allogenic stem cell transplantation. In the AZA-001 trial, the pivotal Phase 3 trial reported in the year 2009, the azacitidine treated group has shown an overall response rate (PR or better) of 29% with a median OS of 24.5 months 6. On the contrary, several other clinical studies, investigating the efficacy of azacitidine in combination with other agents such as lenalidomide, panobinostat, pracinostat, vorinostat etc. reported an overall response rate (PR or better rate) within the range of 28 - 47% and median OS of only 12 - 19 months 12-15. The higher median OS observed in AZA-001 trial might have been due to differences in the patient population enrolled in the study. Nevertheless, for a given response rate, the OS from the AZA-001 trial was considerably higher compared to other studies in our database. Therefore, our meta-analysis suggests that the future clinical trials investigating novel therapeutic agents in combination with azacitidine should include an azacitidine only control arm for direct comparison of the response rates or median OS from combination therapy arm.

Our finding of significantly higher median OS for azacitidine cohorts compared to other cohorts is in agreement with the literature 17, 18. Notably, among the several classes of drugs that were evaluated, azacitidine appears to achieve a longer OS compared to other drugs (such as decitabine) for a given response rate. One possible mechanism for longer OS with azacitidine could be the ability to administer it for a longer period compared to decitabine (median treatment cycles: azacitidine - 6 to 8 cycles; decitabine - 2 to 5 cycles) 16. We also found that the prior treatment status has also a significant impact on median OS. The treatment naïve patients showed higher median OS compared to that of previously treated patients at a given PR or better rate, which is in agreement with the fact that MDS patients with prior treatment or hypomethylating agent failure show poor prognosis and are at higher risk for progression to AML or death 19. This also indicates that there is an urgent need to develop novel therapies for HR-MDS patients who are resistant to the current treatment options.

One of the limitations of this analysis is that 35% of the cohorts included in this analysis had at least 30% AML patients and the conclusions from this analysis may not be generalized to HR-MDS patients (58% of the cohorts had 100% HR-MDS patients) exclusively at a population level. However, one should also note that progression of HR-MDS to AML is very common (30% of HR-MDS patients progress to AML) and including such cohorts (containing both HR-MDS and AML patients) in our analysis was important to ensure that our results are applicable to the real clinical scenario 20. Additionally, we found that population (< 30% AML patients vs ≥ 30% AML patients) was not a significant covariate for the relationship between PR or better rate and median OS, indicating that it is reasonable to combine cohorts with and without the AML patients. In this study, we also combined cohorts evaluated by different response criteria IWG 2000 (24% of the cohorts) vs IWG 2006 (76% of the cohorts) as IWG criteria was not found to be significant covariate for determining the relationship between response rates and median OS. Finally, it was not possible to evaluate the effect of median baseline percent bone marrow blasts on the median OS, as this information was not reported for 82% of the cohorts. However, all the cohorts included in the analysis had patients with IPSS score greater than > 1.5, suggesting that these cohorts are homogenous with respect to the distribution of median baseline percent bone marrow blasts.

The cohorts included in the meta-analysis had similar inclusion-exclusion criteria based on age (70 ± 4 yrs.), sex (%Male = 64 ± 8) and the proportion of high risk MDS patients (80 ± 20%). Despite selecting homogenous cohorts based on age, sex and severity of disease, we observed a wide range of results across the cohorts (median OS: 3.1 - 24.5 months). Hence, for determining the relationship between median OS and response rates, we used a fixed effects model after adjusting for covariates: treatment (AZA vs Other) and prior therapy status (NAÏVE vs Previously treated), which explained 31% and 22% of the variability in median OS respectively. As mentioned in the literature, a random effects model (which requires estimation of between study variability) could be misleading as the results of larger cohorts are somewhat different than that of smaller cohorts 23. Hence, we selected fixed effects model weighted by sample size (rather than commonly used inverse variance weighting for continuous data and Mantel-Haenszel for binary data in random effects model setting) where the point estimate of median OS is simply a weighted average and which doesn't require the homogeneity of variance across the cohorts assumption 23. However, it is worth mentioning that the use of fixed effects model limits the generalizability of the results to a wider MDS population and is applicable to only cohorts like the ones included in this study 23. Additionally, based on this study, one should exercise caution in interpreting the results of cohorts with high response rates (beyond 55% PR or better rate) due to sparse data in this region, which also suggests that there is a need to develop better treatment options with higher response rates leading to prolonged overall survival of HR-MDS patients (Figures 2 - 4).

In summary, the relationship between response rate outcomes and median OS was determined in elderly HR-MDS patients. The analysis demonstrated that PR or better rate was a strong predictor of median OS and that the median OS was longer with azacitidine treatment compared to treatment with other drugs for a given response rate. We also have shown that the median OS of treatment naïve HR-MDS patients was longer compared to that of previously treated patients. These estimated relationships in HR-MDS highlights the potential for PR or better rate, as a potential marker of median OS and may be used to guide decisions on long-term survival using only short-term response rates in the development of new therapies for HR-MDS.

## Figures and Tables

**Table 1:** Summary of the characteristics of the cohorts included in the meta-analysis

Attribute	Number of cohorts (%)
**Total number of cohorts**	38 (100%); Total N = 2400
**Publication Year**	
2008 - 2011	13 (34.2%)
2012-2014	07 (18.4%)
2015-2017	18 (47.4%)
**Cohort Size**	
Median (Range)	45 (11 - 282)
**Age**	
Mean ± SD	70 ± 4 yrs.
Imputed median value	70 yrs.
Missing arms	01 (2.6%)
**% Male**	
Mean ± SD	64 ± 8%
Imputed median value	64%
Missing arms	07 (18.4%)
**% Bone marrow blast**	
Median value (Range)	15% (13-25%)
Missing arms	31 (81.5%)
**Treatment**	
Azacitidine Only	09 (23.7%)
Azacitidine + Other drugs	07 (18.4%)
Decitabine	05 (13.2%)
Lenalidomide	06 (15.8%)
Other drugs/combinations (Cytarabine, Vorinostat etc.)	11 (28.9%)
**Prior treatment Status**	
Hypomethylating agent failure	06 (15.8%)
Prior therapy with other agents	16 (42.1%)
Treatment naïve	16 (42.1%)
**Population**	
< 30% AML patients 100% high risk MDS patients	26 (65%) 22 (58%)
≥ 30% AML patients	14 (35%)
**IWG Criteria**	
Year 2000*	09 (23.7%)
Year 2006**	29 (76.3%)

*CR: ≤ 5% myeloblasts, Hgb ≥ 11 g/dL, Platelets ≥ 100 X 10^9^/L, Neutrophils: 1.5 X 10^9^/L or more, Blasts 0%, No dysplasia; **mCR:** Not defined; **PR:** Same as CR except myeloblasts decreased by ≥ 50% over pretreatment but still > 5% 21. **CR: ≤ 5% myeloblasts, Hgb ≥ 11 g/dL, Platelets ≥ 100 X 10^9^/L, Neutrophils: 1.0 X 10^9^/L or more, Blasts 0%, persistent dysplastic changes will be noted; **mCR:** ≤ 5% myeloblasts and decrease by ≥ 50% over pretreatment; **PR:** Same as CR except myeloblasts decreased by ≥ 50% over pretreatment but still > 5% 22.

## Abbreviations

CR: complete response
mCR: marrow complete response
PR: partial response
HI: Hematological improvement
OS: overall survival
MDS: myelodysplastic syndrome
US FDA: United States Food and Drug Administration
PFS: progression free survival
NCCN: National Comprehensive Cancer Network
